# Mir-302a/TWF1 Axis Impairs the Myogenic Differentiation of Progenitor Cells through F-Actin-Mediated YAP1 Activation

**DOI:** 10.3390/ijms24076341

**Published:** 2023-03-28

**Authors:** Mai Thi Nguyen, Wan Lee

**Affiliations:** 1 Department of Biochemistry, Dongguk University College of Medicine, 123 Dongdae-ro, Gyeongju 38066, Republic of Korea; 2 Channelopathy Research Center, Dongguk University College of Medicine, 32 Dongguk-ro, Ilsan Dong-gu, Goyang 10326, Republic of Korea

**Keywords:** miR-302a, twinfilin-1, myogenesis, proliferation, differentiation, YAP1

## Abstract

Actin cytoskeleton dynamics have been found to regulate myogenesis in various progenitor cells, and twinfilin-1 (TWF1), an actin-depolymerizing factor, plays a vital role in actin dynamics and myoblast differentiation. Nevertheless, the molecular mechanisms underlying the epigenetic regulation and biological significance of TWF1 in obesity and muscle wasting have not been explored. Here, we investigated the roles of miR-302a in TWF1 expression, actin filament modulation, proliferation, and myogenic differentiation in C2C12 progenitor cells. Palmitic acid, the most prevalent saturated fatty acid (SFA) in the diet, decreased the expression of TWF1 and impeded myogenic differentiation while increasing the miR-302a levels in C2C12 myoblasts. Interestingly, miR-302a inhibited TWF1 expression directly by targeting its 3′UTR. Furthermore, ectopic expression of miR-302a promoted cell cycle progression and proliferation by increasing the filamentous actin (F-actin) accumulation, which facilitated the nuclear translocation of Yes-associated protein 1 (YAP1). Consequently, by suppressing the expressions of myogenic factors, i.e., MyoD, MyoG, and MyHC, miR-302a impaired myoblast differentiation. Hence, this study demonstrated that SFA-inducible miR-302a suppresses TWF1 expression epigenetically and impairs myogenic differentiation by facilitating myoblast proliferation via F-actin-mediated YAP1 activation.

## 1. Introduction

Skeletal muscle is one of the most abundant and versatile tissues, and it is primarily responsible for locomotion, energy storage, and metabolism [1]. Skeletal muscle homeostasis is maintained by a sequential process called myogenesis, which is necessary for myofiber development, growth, and regeneration [2]. Skeletal myogenesis is a well-ordered, coordinated process consisting of progenitor cell proliferation, myogenic regulatory factor activation, differentiation, and multinucleated myotube formation [3]. Accordingly, impaired myogenesis provokes muscle wasting, which is characterized by progressive loss of muscle strength and mass [4]. In the past few decades, several lines of evidence have suggested that an excessive intake of saturated fatty acids (SFA) provokes lipotoxicity, as evidenced by oxidative stress, mitochondrial dysfunction, and apoptosis, and these cellular events are known to be closely associated with muscle wasting [5,6]. Nonetheless, the mechanism involved in the impairment of myogenic differentiation triggered by SFA remains undetermined.

Cytoskeleton dynamics, such as assembly, disassembly, rearrangement, and turnover, are coordinated and essential processes necessary for the appropriate activation of diverse myogenic transcription programs in progenitor cells [7,8,9]. The abilities of actin to polymerize and depolymerize to provide mechanical support, structural changes, and cell movement are critical components of cytoskeletal dynamics [9,10]. Several studies have recently demonstrated that actin-binding proteins (ABPs) can orchestrate mechanotransduction, a multistage signal transduction pathway that senses and translates mechanical changes into biochemical signals, thereby regulating the cellular responses associated with the cell cycle and proliferation [9,10,11,12,13]. For example, MPRIP, as an ABP, has been recently found to regulate cellular responses associated with the cell cycle and proliferation by F-actin and transcriptional modulation of the cells [14,15]. By modulating the transitions between globular actin (G-actin) and filamentous actin (F-actin), ABPs function as critical regulators of Yes-associated protein 1 (YAP1) during mechanotransduction [16,17,18]. YAP1 is a transcriptional coactivator of the Hippo signaling pathway, which regulates multiple biological processes, such as cell cycle progression, proliferation, differentiation, migration, and survival [19]. Therefore, it was suggested that F-actin deposition stimulates cell proliferation and hinders myogenic differentiation via mechanotransduction-mediated YAP1 activation [20]. We previously demonstrated that twinfilin-1 (TWF1), an actin filament depolymerizing factor, is indispensable for the differentiation of myogenic progenitor cells by regulating F-actin, YAP1, and cell proliferation [21]. However, the role played by TWF1 in the pathogenesis of muscle wasting, particularly in obesity, is largely unknown.

MicroRNAs (miRNAs) are a class of non-coding RNAs that regulate gene expression epigenetically in most biological processes by binding to the 3′UTR of target mRNAs [22]. MiRNAs are also known to be crucial regulators of cell proliferation, cell cycle, and differentiation during myogenesis [23,24,25,26,27], and many studies indicate that dysregulations of specific miRNAs underlie the pathophysiology of myopathies [28,29]. In addition, several miRNAs altered by SFA and obesity have been linked to oxidative stress, mitochondrial dysfunction, and ER stress in the context of muscle wasting [30,31,32]. Previously, the miR-302/367 cluster, which consists of five members, has been reported to play numerous roles in cellular processes as diverse as proliferation, differentiation, and reprogramming [33]. Of these, miR-302a-3p (miR-302a) is upregulated in oxidative stress and mitochondrial dysfunction, which are both causally linked to muscle wasting [34]. Moreover, upregulation of miR-302a was reported in the livers of diet-induced obese mice [35] and HepG2 cells treated with palmitic acid (PA), the most abundant SFA in the diet [36], indicating an association between miR-302a and SFA or diet-induced obesity. Interestingly, miR-302a has been implicated in the suppression of neuronal cell differentiation [37,38], and a bioinformatic analysis of the miRNA–mRNA interaction using a TargetScan analysis suggested that TWF1 is a potential target of miR-302a. Nonetheless, the significance and mechanism of miR-302a in the regulation of TWF1 expression and myogenic differentiation have not been investigated.

In this study, we revealed that PA inversely regulated the expression of TWF1 and miR-302a in myogenic progenitor cells and that TWF1 is a direct target of miR-302a. As TWF1 is a crucial player in myoblast differentiation and myotube formation, we investigated the effect of miR-302a on myogenic factor expression, differentiation, and the myotube formation of progenitor cells. Furthermore, we demonstrated the mechanisms responsible for YAP1 activation, cell cycle progression, myoblast proliferation, and myogenic differentiation by miR-302a. Thus, this study elucidated the role of miR-302a in the regulation of TWF1 and myogenesis and suggested a possible mechanism for miRNA-mediated muscle wasting in obesity.

## 2. Results

### 2.1. PA Suppresses TWF1 but Increases miR-302a Expression in Myoblasts

**Figure 1:**
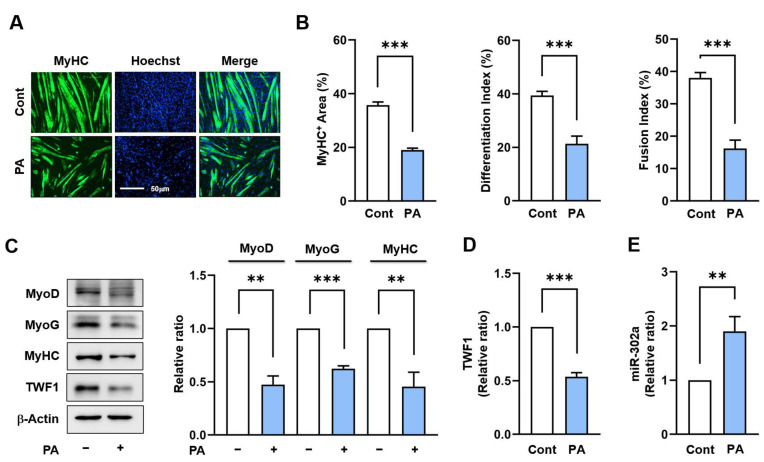
PA impairs myogenic differentiation and modulates TWF1 and miR-302a expression. C2C12 myoblasts were treated with PA (100 μM) or a vehicle for 24 h and differentiated for 5 days. (**A**) Immunofluorescence staining for MyHC (MF20, green) and nucleus (Hoechst 34452, blue) on differentiation day 5. Scale bar: 50 μm. (**B**) Myogenic differentiation was analyzed using MyHC-positivity areas, differentiation and fusion indices as described in the Materials and Methods. (**C**,**D**) The expression levels of myogenic factors (MyoD, MyoG, and MyHC) and TWF1 were determined by immunoblots on differentiation day 3 and normalized versus β-actin by densitometry. (**E**) The expression levels of miR-302a were analyzed by RT-*q*PCR after 24 h of PA treatment and normalized versus U6. Results are expressed as relative ratios versus vehicle controls and presented as means ± standard errors (*n* > 3); **, *p* < 0.01; ***, *p* < 0.001 vs. controls.

To determine the effect of PA on the expression of TWF1 and miR-302a, C2C12 cells were treated with PA (100 μM), which is known to impair myotube formation [39], for 24 h and then allowed to differentiate for five days. Immunocytochemistry using the myosin heavy chain (MyHC) antibody showed that cells treated with PA produced shorter and fewer MyHC-positive myotubes containing fewer nuclei than cells treated with a vehicle (Figure 1A,B). In addition, PA-treated C2C12 cells exhibited marked reductions in the levels of the myogenic regulatory factors, i.e., MyoD and MyoG, as determined on day 3 of the differentiation (Figure 1C). These results were consistent with our previous investigation showing that PA decreases the expression of myogenic factors and inhibits the formation of myotubes [39]. Under this experimental condition, we next assessed whether PA affects the expression levels of TWF1 and miR-302a in C2C12 cells, because it has been reported that TWF1 is required for myogenic differentiation [21] and miR-302a is upregulated in obese mice and PA-treated HepG2 cells [35,36]. Indeed, PA reduced the TWF1 protein levels by approximately 60% but increased the miR-302a levels about two-fold (Figure 1C,D). These results indicate that PA inhibits myogenic differentiation, downregulates TWF1, and upregulates miR-302a expression in C2C12 myoblasts.

### 2.2. TWF1 Is Directly Targeted by miR-302a

**Figure 2:**
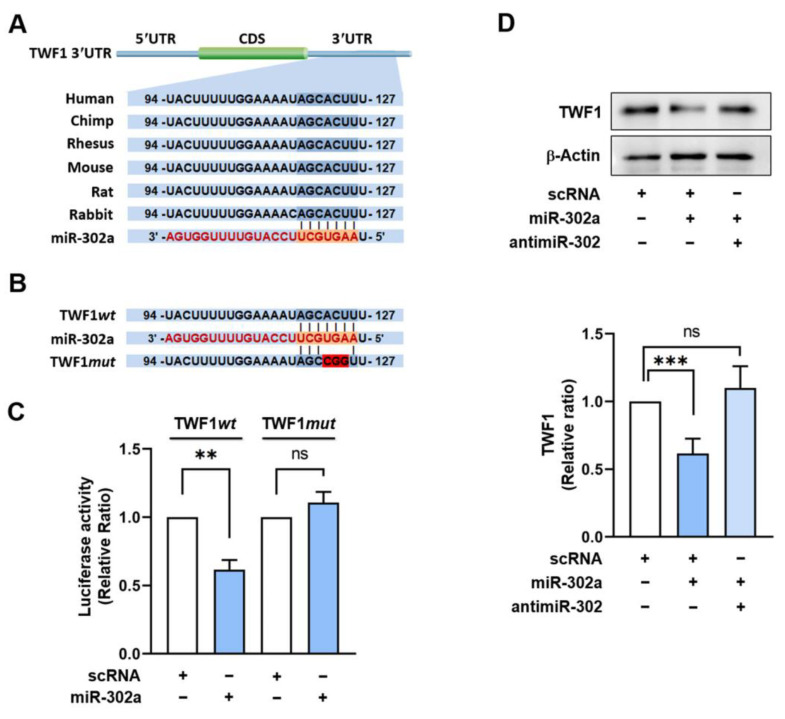
MiR-302a suppresses TWF1 expression by directly binding to *TWF1* 3′UTR. (**A**) Schematic diagram showing the potential miR-302a binding site on the 3’UTR of *TWF1*. (**B**) Sequences of the miR-302a binding sites of wild-type (TWF1*wt*) and mutant (TWF1*mut*) *TWF1* 3’UTR. (**C**) The dual-luciferase reporter assay was conducted 24 h after transfection in C2C12 cells. (**D**) Representative immunoblots of TWF1 in C2C12 cells 24 h after transfection with scRNA or miR-302a mimic. Immunoblot results are expressed as relative ratios versus scRNA controls and presented as means ± standard errors (*n* > 3); **, *p* < 0.01; ***, *p* < 0.001 vs. scRNA. ns: no significance.

As the expressions of TWF1 and miR-302a are inversely related in PA-treated C2C12 cells, we investigated whether miR-302a suppresses TWF1 expression by directly binding to its 3′UTR. The potential target of miR-302a was explored using miRNA target prediction algorithms, such as PicTar and TargetScan. Interestingly, we found that the 3′UTR of *TWF1* mRNA contains a highly conserved binding region for miR-302a located from nucleotides 119 to 125, which is evolutionarily conserved in humans, mice, and rabbits (Figure 2A). To confirm whether the 3’UTR of *TWF1* contains an authentic binding site for miR-302a, we cloned a *TWF1* 3′UTR containing the miR-302a seed binding site (wild type; TWF1*wt*) or a mutant *TWF1* 3′UTR (TWF1*mut*) with three base mutations in the binding site to a pmirGLO dual-luciferase reporter plasmid (Figure 2B). The reporter constructs were cotransfected with miR-302a mimic or scRNA into C2C12 cells, and the luciferase activity was measured 24 h later. As was expected, the luciferase activity of the TWF1*wt* construct was remarkably reduced by the miR-302a mimic as compared with the scRNA control (Figure 2C). In contrast, the luciferase activity of TWF1*mut* was unaffected, demonstrating direct binding between the 3′UTR of *TWF1* and miR-302a (Figure 2C). Next, to confirm that TWF1 is an authentic target of miR-302a, we transfected cells with the miR-302a mimic for 24 h and assessed the endogenous TWF1 protein levels. The results showed that the miR-302a mimic decreased the protein level of TWF1 significantly versus the scRNA controls (Figure 2D). Furthermore, inhibition of the miR-302a mimic using a 2’-O-methyl antisense inhibitor of miR-302a (antimiR-302) completely prevented the miR-302a mimic-induced reduction in TWF1 protein expression (Figure 2D). Overall, these results suggest that miR-302a negatively regulates TWF1 expression by directly binding to the 3′UTR of *TWF1*.

### 2.3. MiR-302a Increases F-Actin Accumulation and the Nuclear Translocation of YAP1

**Figure 3:**
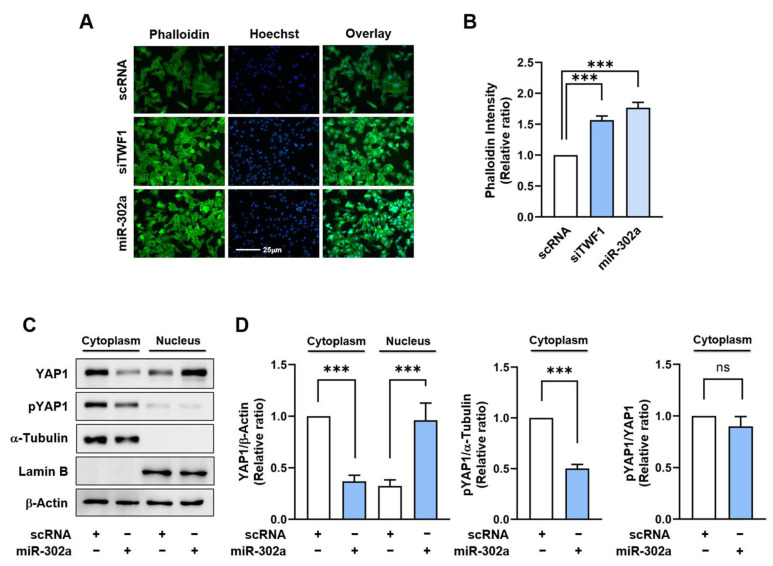
MiR-302a promotes F-actin formation and the nuclear localization of YAP1. C2C12 cells were transfected with scRNA, siTWF1, miR-302a mimic, or antimiR-302 for 24 h before analysis. (**A**,**B**) F-actin was stained with FITC-phalloidin (green). Nuclei were counterstained with Hoechst 34452 (blue). Scale bar: 25 μm. (**C**,**D**) Immunoblots of YAP1 and phosphorylated YAP1 (pYAP1) in the nuclear and cytoplasmic fractions. α-Tubulin and Lamin B were applied as cytoplasmic and nuclear fraction markers, respectively. Immunoblot results are expressed as relative ratios versus Lamin B or α-Tubulin and presented as means ± standard errors (*n* > 3); ***, *p* < 0.001 vs. scRNA. ns: no significance.

Since it has been demonstrated that TWF1 knockdown in myoblasts promotes cell proliferation by increasing the F-actin and nuclear YAP1 levels [21], we explored whether miR-302a overexpression results in F-actin accumulation and the nuclear translocation of YAP1. The levels of F-actin in the cells transfected with the miR-302a mimic or TWF1 siRNA (siTWF1) were significantly elevated by 50% versus the scRNA controls (Figure 3A,B), and this enhancement was attributed to inefficient actin depolymerization resulting from TWF1 depletion because the immunoblot analysis showed no difference in the total actin levels between the groups. This finding supports the hypothesis that miR-302a impedes actin depolymerization through TWF1 suppression and consequently increases F-actin accumulation. Recently, actin stress fibers have been demonstrated to inhibit the phosphorylation of the transcriptional coactivator YAP1 [40,41]. Phosphorylation of YAP1 at serine residues 127 and 381 results in its sequestration in the cytoplasm and subsequent proteasomal degradation, while dephosphorylation of these sites promotes its translocation to the nucleus and activation of the transcriptional factors that promote cell proliferation [40,41]. Hence, we next assessed the phosphorylation (S127) and nuclear levels of YAP1 to determine whether miR-302a facilitates its nuclear translocation. Transfection of the miR-302a mimic in the myoblasts caused a significant decline in the phosphorylated YAP1 levels in the cytoplasm and increased the nuclear YAP1 levels (Figure 3C,D), which confirmed miR-302a can stimulate the nuclear translocation of YAP1 from the cytoplasm to the nucleus.

### 2.4. MiR-302a Increases Myoblast Proliferation

**Figure 4:**
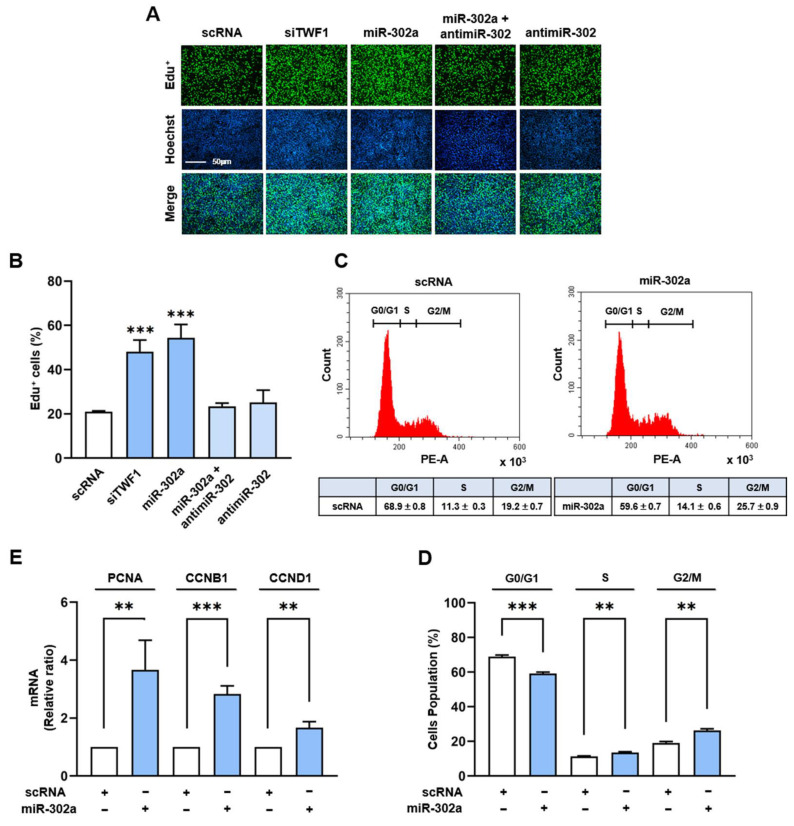
MiR-302a promotes myoblast proliferation. C2C12 cells were transfected with scRNA, siTWF1, miR-302a mimic, or antimiR-302 for 24 h before analysis. (**A**) EdU incorporation analysis. Cells undergoing DNA replication were labeled with EdU (green) and nuclei were counterstained with Hoechst 34452 (blue). Scale bar: 50 μm. (**B**) Proportions of EdU-positive cells were determined using ImageJ software. (**C**,**D**) Representative flow cytometry results. The scatter plots of cell cycle images and cell cycle phase proportions were obtained after 24 h of transfection. (**E**) Relative expression levels of CCNB1, PCNA, and CCND1 were determined via RT-*q*PCR and normalized versus U6. *q*RT-PCR results are expressed as relative ratios versus controls (scRNA) and presented as means ± standard errors (*n* > 3); **, *p* < 0.01; ***, *p* < 0.001 vs. scRNA.

Given that an increase in the nuclear YAP1 level enhances cell cycle progression and proliferation [42,43,44], we next examined whether the induction of miR-302a would promote cell cycle progression and proliferation in C2C12 myoblasts. The cells were transfected with the miR-302a mimic, siTWF1, antimiR-302, or scRNA control, and a cell proliferation assay was performed using EdU, as described in the Materials and Methods. Transfection with siTWF1 increased the EdU incorporation into the myoblasts by approximately 2.5-fold versus the scRNA controls (Figure 4A,B). Similarly, transfection with the miR-302a mimic in the myoblasts markedly enhanced the EdU incorporation by more than three-fold, whereas cotransfection with antimiR-302 and the miR-302a mimic almost entirely abrogated the effect of miR-302a on EdU incorporation (Figure 4A,B), suggesting that miR-302a stimulates myoblast proliferation. In addition, we evaluated the effect of miR-302a on the cell cycle via flow cytometry. Transfection of the miR-302a mimic increased the percentage of cells in the S and G2/M phases and reduced the percentage of cells in the G0/G1 phase (Figure 4C,D), indicating that miR-302a promotes cell cycle progression. To support the observed effects of miR-302a on cell cycle progression and cell proliferation, the transcript levels of the cell cycle-related genes, i.e., CCNB1, CCND1, and PCNA, were analyzed in C2C12 myoblasts by means of *q*RT-PCR. In line with our cell cycle results, the mRNA levels of these genes were significantly higher in the cells transfected with the miR-302a mimic than in those transfected with scRNA (Figure 4E). These findings show that miR-302a promotes myoblast proliferation and cell cycle progression.

### 2.5. MiR-302a Suppresses Myogenic Factor Expressions

**Figure 5:**
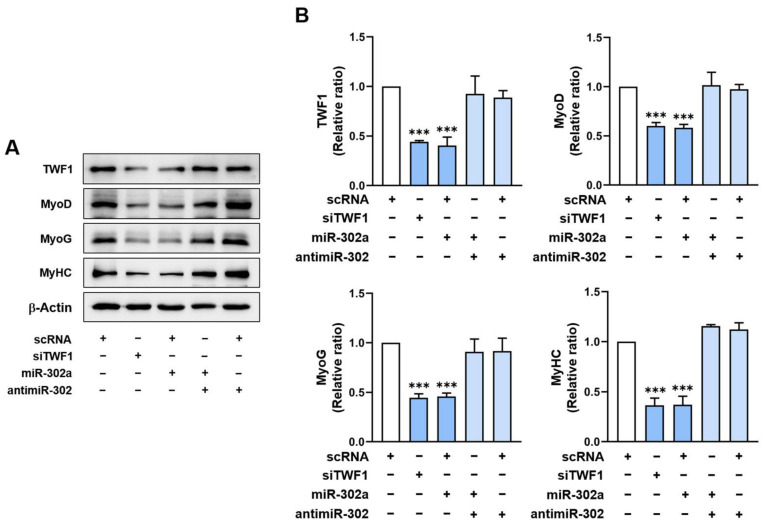
MiR-302a inhibits the expressions of myogenic factors and TWF1. C2C12 cells were transfected with scRNA, siTWF1, miR-302a mimic, or antimiR-302 and differentiated for 3 days. (**A**) Representative immunoblots of MyoD, MyoG, MyHC, and TWF1 with β-actin are shown. (**B**) Quantitative analysis of TWF1 and myogenic factor (MyHC, MyoD, and MyoG) protein levels. Intensities of immunoblots were normalized versus β-actin via densitometry. Values are relative ratios versus controls (scRNA). Results are presented as means ± standard errors (*n* > 3); ***, *p* < 0.001 vs. scRNA.

As myoblast proliferation and differentiation are inversely related during myogenesis [3] and miR-302a was found to facilitate myoblast proliferation, we next investigated whether miR-302a suppresses the expressions of myogenic regulatory factors. C2C12 myoblasts were transfected with scRNA, siTWF1, miR-302a mimic, or antimiR-302, and the expressions of the myogenic factors were assessed on day 3 of differentiation. Transfection of C2C12 cells with siTWF1 decreased the TWF1 protein levels by 55% and dramatically reduced the expressions of MyoD and MyoG as compared with the scRNA controls (Figure 5A,B). It is noteworthy that miR-302a mimic transfection significantly repressed the TWF1, MyoD, and MyoG expressions compared to the scRNA controls. In addition, cotransfection with the miR-302a mimic and antimiR-302 dramatically eliminated the suppressive effect of the miR-302a mimic on the expression of TWF1 and the myogenic regulatory factors (Figure 5A,B). Based on the bioinformatic analysis results, the 3’UTRs of MyoD, MyoG, and MyHC do not contain any tentative binding sites for miR-302a or antimiR-302. Thus, the inhibition of myogenic factor expression by the miR-302a mimic is attributed mainly to the reduction of TWF1 expression.

### 2.6. MiR-302a Impairs Differentiation and Myotube Formation of Myoblasts

**Figure 6:**
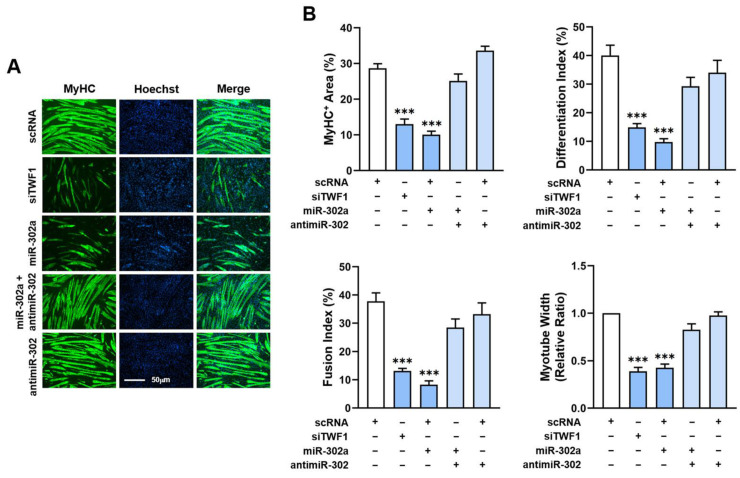
MiR-302a impairs myogenic differentiation. C2C12 cells were transfected with scRNA, siTWF1, miR-302a mimic, or antimiR-302 and differentiated for 5 days. (**A**) Immunofluorescence staining for MyHC (MF20, green) and nucleus (Hoechst 34452, blue) on differentiation day 5. Scale bar: 50 μm. (**B**) Myogenic differentiation was analyzed using MyHC-positivity areas, differentiation and fusion indices, and myotube widths. Values are relative ratios versus controls (scRNA). Results are presented as means ± standard errors (*n* > 3); ***, *p* < 0.001 vs. scRNA.

Since miR-302a inhibited the expressions of the myogenic regulatory factors, we investigated whether miR-302a modulates myoblast differentiation and myotube formation. Myoblasts were transfected with the specified oligonucleotides and cultured in DM for five days to induce myogenic differentiation (Figure 6A,B). The differentiation and myotube formation were analyzed via immunocytochemistry using the MyHC antibody and an image analyzer. TWF1 depletion markedly impeded the myogenic differentiation as assessed using the MyHC-positive areas, differentiation and fusion indices, and myotube widths (Figure 6A,B). Notably, transfection with the miR-302a mimic significantly reduced the MyHC-positive area proportions, and the numbers of nuclei and myotube sizes revealed that the miR-302a mimic generated myotubes with fewer than six nuclei and a lower differentiation index (Figure 6A,B). To confirm the role of miR-302a in myogenic differentiation, myoblasts were cotransfected with antimiR-302 or scRNA. As was expected, antimiR-302 dramatically abolished the inhibitory effect of the miR-302a mimic on myoblast differentiation and myotube formation (Figure 6A,B). These results clearly demonstrate that miR-302a impairs the myogenic differentiation of C2C12 myoblasts.

## 3. Discussion

A growing body of evidence suggests that miRNAs play critical roles in the development and regeneration of skeletal muscles [28,29]. Furthermore, the recent discovery of coordination between cytoskeleton dynamics and mechanotransduction has improved our understanding of the significance of miRNAs targeting ABPs in skeletal myogenesis. This study reveals the roles played by SFA-inducible miR-302a in TWF1 expression, F-actin modulation, cell proliferation, and the myogenic differentiation of progenitor cells. Specifically, the following findings provide novel insights that extend existing knowledge: (i) PA suppresses TWF1 expression while increasing miR-302a expression in C2C12 myoblasts; (ii) MiR-302a inhibits TWF1 expression by targeting the 3’UTR of *TWF1* directly; (iii) MiR-302a augments F-actin and enhances the nuclear YAP1 levels, and hence it promotes cell proliferation; and (iv) MiR-302a inhibits myogenic factor expression and the myogenic differentiation of progenitor cells. Collectively, SFA-inducible miR-302a reduces TWF1 expression and impairs myogenic differentiation via F-actin-mediated YAP1 activation.

Although SFA are known to inhibit the differentiation of diverse types of progenitor cells [5,45], it remains undetermined how miRNAs affected by SFA or obesity impair the differentiation of progenitor cells. This study suggests that a reduction in TWF1 expression underlies myogenic differentiation impairment by SFA, and this may be an etiology of muscle wasting in obesity. TWF1 is a highly conserved cytoplasmic ABP belonging to the actin-depolymerizing factor family [46]. Due to its ability to cap the barbed ends of F-actin and the sequester monomers of G-actin, TWF1 protein is critically required for actin cytoskeletal dynamics [47,48,49,50]. Accordingly, TWF1 induction lowers the F-actin levels by enhancing filament depolymerization [51], whereas TWF1 knockdown increases F-actin accumulation, thereby exacerbating cytoskeletal aberrations [47,48,49,50]. In accordance with our previous findings [21], the present study shows that TWF1 knockdown by siTWF1 stimulated the nuclear translocation of YAP1 and subsequently promoted the proliferation of myogenic progenitor cells (Figure 3 and Figure 4). Furthermore, TWF1 knockdown consequently suppressed the myogenic transcription factor expressions and the differentiation and myotube formation in myoblasts (Figure 5 and Figure 6). As TWF1 reduction is regarded as a primary factor in the SFA-induced suppression of myogenic differentiation, it would appear that inhibition of TWF1 expression in myoblasts contributes to sarcopenic obesity by linking fat accumulation with muscle wasting.

The current study also revealed the mechanism responsible for TWF1 suppression by SFA in myoblasts. Before undertaking this study, we focused on the epigenetic regulation of TWF1 expression by miR-302a, since miR-302a expression is affected by SFA and TWF1 was suggested to be a tentative target gene of miR-302a by a bioinformatic target prediction analysis (Figure 2). As a member of the miR-302/367 cluster, miR-302a has been implicated in a variety of biological processes, including cell proliferation, differentiation, and regeneration [33]. Furthermore, it should be noted that the expression of miR-302a was found to be significantly upregulated in myocardial apoptosis, mitochondrial damage, and oxidative stress induced by myocardial ischemia-reperfusion injury in mice [34], because these conditions are associated with the etiology of muscle wasting [30,31,32]. Moreover, we observed that PA significantly increased miR-302a expression in myoblasts (Figure 1), which suggested that PA alone might elevate miR-302a expression in myoblasts. However, the mechanism responsible for the induction of miR-302a by SFA requires further study. Nevertheless, the present study shows that miR-302a suppresses TWF1 expression directly by targeting the 3′UTR of *TWF1* in myoblasts. Few studies have demonstrated the significance of miRNAs in ABP expression and myogenic differentiation; hence, this study is the first to report the epigenetic regulation of the actin-depolymerizing protein TWF1 by a specific miRNA. These results also support a molecular mechanism that results in impaired myogenic differentiation mediated by a miRNA that targets TWF1.

Myogenesis is regulated by an inverse relationship between myoblast proliferation and differentiation into myotubes, and therefore, cell cycle exit is a prerequisite for myoblast differentiation [3]. In this respect, the suppression of myogenic differentiation by miR-302a may be primarily ascribed to increased myoblast proliferation. Then, how does miR-302a promote cell cycle progression and proliferation in myoblasts? Cell proliferation and myogenic transcriptional activation in progenitor cells require precisely orchestrated cytoskeletal dynamics, such as assembly, disassembly, and cytoskeletal rearrangement [7,8,9]. With regard to the mechanism of mechanotransduction, the actin cytoskeleton plays a crucial role in the Hippo signaling pathway by regulating YAP1 [52]. The phosphorylation of YAP1 mediated by Hippo kinase in this signaling pathway has been shown to inhibit the nuclear translocation of YAP1 by enhancing its proteasomal degradation in the cytoplasm [53]. On the other hand, F-actin accumulation in cytoplasm suppresses Hippo kinase activity, which reduces YAP1 phosphorylation, resulting in the nuclear translocation of YAP1 and consequently enhancing cell proliferation and cell cycle progression [40,42,43,44]. Therefore, TWF1 knockdown increases the formation of F-actin and the nuclear localization of YAP1, and it also increases cell proliferation and cell cycle progression [18]. Collectively, miR-302a induction increased F-actin formation by suppressing TWF1 expression (Figure 3), facilitated the nuclear translocation of YAP1 and the transcriptional activation of genes involved in cell proliferation (Figure 4), and ultimately, impaired myogenic factor expression and differentiation (Figure 5 and Figure 6).

## 4. Materials and Methods

### 4.1. Cell Culture and PA Treatment

The C2C12 cells, a murine skeletal muscle cell line (ATCC, Manassas, VA, USA), were cultured in a growth medium (GM; DMEM supplemented with 10% fetal bovine serum (FBS) and 1% penicillin/streptomycin) (Gibco, Carlsbad, CA, USA) in a 5% CO_2_ atmosphere at 37 °C. To induce differentiation, the GM was changed to a differentiation medium (DM; DMEM containing 2% horse serum and 1% penicillin/streptomycin) (Gibco) when the cell confluency reached 80–90%. The bovine serum albumin (BSA)-conjugated PA was prepared and stored at −80 °C, as previously described [54]. When necessary, the C2C12 cells were pretreated with PA (100 µM) or a vehicle (control) for 24 h in GM.

### 4.2. Transfection of Oligonucleotides

The synthesized oligonucleotides viz. TWF1 siRNA (siTWF1), miR-302a mimic, miR-302 inhibitor (anti-miR-302), and scrambled control RNA (scRNA) were purchased from Genolution (Seoul, Korea). The oligonucleotide sequences are presented in Table S1. The C2C12 cells were grown in GM for the oligonucleotide transfection (1.3 × 10^5^ cells/35 mm plates) or plasmid transfection (1 × 10^4^ cells/12-well plates). When 30–40% confluent, the cells were transfected with the indicated oligonucleotides or plasmids using Lipofectamine 2000 (Invitrogen, Waltham, MA, USA) in FBS-free DMEM, and the medium was replaced with GM 4 h after transfection.

### 4.3. Dual-Luciferase Reporter Assay

The 3′UTR of TWF1 containing the potential miR-302a binding site was synthesized from mouse cDNA using Pfu DNA polymerase (Invitrogen) and inserted into the pmirGLO vector to generate a wild-type plasmid (TWF1*wt*). The site-directed mutagenesis was carried out using overlapping PCR containing the mutated target sequence of miR-302a and cloned into the pmirGLO vector to generate a mutated plasmid (TWF1*mut*). The primers and PCR conditions used for the cloning and mutation are listed in Table S2. The cells were grown in a 12-well plate (5 × 10^4^ cells/well) for 24 h, and the pmirGLO luciferase vector (0.5 μg) containing TWF1*wt* or TWF1*mut* was cotransfected with miR-302a or scRNA (70 nM) into the C2C12 cells as described above. After 24 h of transfection, the luciferase activity was analyzed using a Dual-Luciferase Reporter Assay System 100 kit (Promega, Madison, WI, USA) and the Sirius L Single Tube Luminometer system (Berthold Technology, Bad Wildbad, Germany). The luciferase activities were determined as the Renilla/Firefly luminescence ratios.

### 4.4. RT-qPCR

The total RNA was isolated from the C2C12 cells using the Qiazol reagent and purified with a miRNeasy Mini Kit (Qiagen, Hilden, Germany). The reverse transcription of the extracted RNAs (1 µg) was performed using a PCR Applied Biosystem Instrument (Thermo Fisher Scientific, Waltham, MA, USA) and a miScript II RT Kit (Qiagen). The mRNA levels of specific genes and miRNA were assessed via RT-*q*PCR, which was conducted using iTaq polymerase and SYBR Green I (Promega) in a LightCycler 480 (Roche Applied Science, Penzberg, Germany). The results were calculated using the 2^−ΔΔCt^ method based on U6 as the internal reference. The RT-*q*PCR primer sequences and conditions are presented in Table S2.

### 4.5. Cell Fractionation for Cytoplasm and Nucleus

The cytoplasmic and nuclear extractions were obtained using the NE-PER^TM^ Nuclear and Cytoplasmic Extraction Reagents (Thermo Fisher Scientific) after 24 h of transfection. Briefly, the cells were scraped in the presence of trypsin EDTA (Gibco) and spun down at 3000 rpm (5 min, 4 °C). Following three rounds of washing with pre-chilled PBS, the cell pellets were resuspended in 100 µL CER I and vortex mixed at a maximum speed three times for 10 min. CER II (5.5 µL) was then added to the cell lysates, incubated for 1 min, and centrifuged. The supernatants (cytosolic fractions) and pellets (nucleus) were then collected. The pellets were resuspended and lysed in 50 µL NER solution for 40 min, centrifuged, and the supernatants were collected as nuclear fractions.

### 4.6. Immunoblotting

The total protein was extracted from the transfected cells using a lysis buffer containing 2% Triton-X, 0.2 mM PMSF, and 1% phosphatase inhibitor cocktail II (Sigma-Aldrich, St. Louis, MO, USA). The protein concentrations were assessed via a Bradford assay using a UV-1700 PharmaSpec spectrophotometer (Shimadzu, Kyoto, Japan) at 595 nM. The samples (20 µg/lane) were then loaded on SDS-PAGE, electrophoresed, incubated with specific primary antibodies (Table S3) overnight at 4 °C, and labeled with HRP-conjugated anti-rabbit or anti-mouse IgG antibodies for 1 h. The blots were developed using a Femto commercial kit (Thermo Fisher Scientific) with a Fusion Solo system (Vilber, Marne-la-Vallée, France). The intensities of the blots were analyzed with Evolution Capt software (Vilber).

### 4.7. Immunofluorescence Analysis

Immunostaining was performed on differentiation day 5. Briefly, the cells were fixed with paraformaldehyde (4%, 10 min), permeabilized with Triton X-100 (0.3%, 15 min), blocked with BSA (3%, 2 h), incubated with MyHC antibody (1:100 dilution, 4 °C, overnight), and treated with Alexa 488 fluorescent secondary antibody (Thermo Fisher Scientific) for 1.5 h. For the F-actin staining, the cells were plated in 8-well chamber slides (10^3^ cells/well) for 24 h, fixed, permeabilized, and stained for 40 min with FITC-conjugated phalloidin (P5282, 50 μg/mL, Sigma) in PBS. The nuclei were counterstained by treating the cells with Hoechst 33342 (Invitrogen) for 15 min. Images were captured randomly using a Leica fluorescence microscope (Microsystems, Mannheim, Germany). The measurements were taken from five separate areas for at least three independent experiments. The MyHC-positive areas, myotube widths, and differentiation and fusion indices were determined as previously described [21].

### 4.8. Ethynyl Deoxyuridine (EdU) Assay

The C2C12 cells (10^4^ cells/well) were seeded and transfected with the indicated oligonucleotides in 8-well chamber slides and assayed using a Click-iT™ EdU Cell Proliferation Kit (Invitrogen). After 24 h of transfection, the cells were treated with 10 µM EdU for 4 h in GM at 37 °C. Next, the cells were fixed, permeabilized, and stained with 0.3 mL of Click-iT reaction cocktail for 20 min. The nuclei were stained with Hoechst 33342. Then, the cells were imaged under a Leica fluorescence microscope (Microsystems). To determine the percentage of EdU-positive cells among the total cells, the cells were counted in three randomly chosen fields per experiment in three independent experiments.

### 4.9. Flow Cytometry Assay

The cell cycle distributions were determined using a Cell Cycle kit (C03551, Beckman Coulter, Brea, CA, USA). After harvest, the cells were fixed overnight with 70% ethanol at 4 °C and then incubated in Cell Cycle solution (0.5 mL, 20 min) in the dark. The cell cycle assays were conducted using a CytoFLEX instrument (Beckman Coulter, USA).

### 4.10. Database and Statistical Analysis

The potential binding site of miRNA on the 3′UTR of *TWF1* was predicted using publicly available in silico analysis tools, such as Pictar (pictar.mdc-berlin.de) and TargetScan (www.targetscan.org). The experiments were performed at least three times independently, and the results are presented as means ± standard errors. The statistical analysis was conducted using Student’s *t*-test for unpaired data.

## 5. Conclusions

A reduction in TWF1 expression by PA has been first demonstrated in this study, which suggests that this could contribute to the pathogenesis of sarcopenia in obesity by connecting fat deposition to impaired myogenesis. Moreover, we demonstrated that SFA-inducible miR-302a suppresses TWF1 expression epigenetically and impedes myogenic differentiation by enhancing myoblast proliferation through the F-actin/YAP1 axis. Therefore, the roles of miR-302a in TWF1 expression, F-actin modulation, cell proliferation, and myogenic differentiation provide novel insights into the mechanisms by which SFA regulate actin dynamics and myogenesis.

## Data Availability

The data presented in this study are available on request from the corresponding author.

## Supplementary Materials

The supporting information can be downloaded at https://www.mdpi.com/article/10.3390/ijms24076341/s1.
