# Supplementary material for: Mir-302a/TWF1 Axis Impairs the Myogenic Differentiation of Progenitor Cells through F-Actin-Mediated YAP1 Activation
Source: Int J Mol Sci. 2023 Mar 28;24(7):6341. doi: 10.3390/ijms24076341 (PMC10094299; doi:10.3390/ijms24076341)
Supplement: Supplementary file 1 [file ijms-24-06341-s001.zip › ijms-2304717-supplementary.pdf]

Table S1. Oligonucleotide sequences for transfection

Gene	Primer sequence (5´-3´)
scRNA (control RNA)	UCACAACCUCCUAGAAAGAGUAGA
TWF1 siRNA (siTWF1)	CGUUACCAUUUCUUUCUGUUU
miR-302a	UAAGUGCUUCCAUGUUUUGGUGA
antimiR-302	UCACCAAAACAUGGAAGCACUUA

Table S2. Primer lists and conditions for *q*RT-PCR and cloning

(A) Mouse primer lists for *q*RT-PCR and RT-PCR

Gene	Primer sequence (5´-3´)		Product size	Annealing Temperature	Concentration		Cycle
					cDNA	Primer	
U6	F.P	CTCGCTTCGGCAGCACA	94	58	2 ng/μl	0.5 μM	40
	R.P	AACGCTTCACGAATTTGCGT					
PCNA	F.P	GAACCTGCAGAGCATGGACTC	201	58			
	R.P	GGTGTCTGCATTATCTTCAGCCC					
CCNB1	F.P	GAGCTATCCTCATTGACTGG	125	58			
	R.P	CATCTTCTTGGGCACACAAC					
CCND1	F.P	ACCAATCTCCTCAACGACCG	228	58			
	R.P	ACGGAAGGGAAGAGAAGGG					

(B) Primer lists for wild-type and mutant 3'UTR cloning

Gene	Primer sequence (5´-3´)		Product size	Annealing Temperature	Concentration		Cycle
					cDNA	Primer	
TWF1*wt*	F.P	AGTCCAGCTTTTAGTACAGG	262	58	2 ng/μl	0.5 μM	35
	R.P	TCTAGAACTGGCCCAACT					
TWF1*mut*	F.P	AGTCCAGCTTTTAGTACAGG	97				
	R.P	AGAAGTGAAACCGGCTATTTTCC					
	F.P	GGAAAATAGCCGGTTTCACTTCT	188				
	R.P	TCTAGAACTGGCCCAACT					

Table S3. Antibodies list

Antibody	Type	Targeted species	Manufacturer	Cat. No.	Dilution ratio*
TWF1	Polyclonal	Rabbit	Proteintech, Rosemont, Illinois, USA	11732-1-AP	1:5,000
MyHC	Monoclonal	Mouse	DSHB, Iowa, IA, USA	MF20	1:1,000
MyoD	Monoclonal	Mouse	Santa Cruz Biotechnology, Dallas, TX, USA	sc-377460	1:1,000
MyoG	Monoclonal	Mouse	Santa Cruz Biotechnology, Dallas, TX, USA	sc-12732	1:1,000
YAP1	Monoclonal	Rabbit	Cell Signaling Technology, Danvers, MA, USA	14074S	1:10,000
p-YAP1	Polyclonal	Rabbit	Cell Signaling Technology, Danvers, MA, USA	4911S	1:10,000
Lamin B2	Monoclonal	Rabbit	Abcam, Cambridge, United Kingdom	ab151735	1:2,500
α-Tubulin	Monoclonal	Mouse	DSHB, Iowa, IA, USA	12G10	1:2,000
β-actin	Monoclonal	Rabbit	Sigma-Aldrich Chemical, St. Louis USA	A2066	1:10,000
Antibodies HRP-linked anti-rabbit IgG			Cell Signaling Technology, Danvers, MA, USA	#7074	1:10,000
Goat anti-mouse(H+L)			Invitrogen, Thermofisher Scientific, Waltham, MA, USA	#32430	1:2,000

*All blots were visualized using a Femto reagent (Thermofisher Scientific).
